# Evaluate Pavement Skid Resistance Performance Based on Bayesian-LightGBM Using 3D Surface Macrotexture Data

**DOI:** 10.3390/ma15155275

**Published:** 2022-07-30

**Authors:** Yuanjiao Hu, Zhaoyun Sun, Yuxi Han, Wei Li, Lili Pei

**Affiliations:** School of Information Engineering, Chang’an University, Xi’an 710064, China; sandra@chd.edu.cn (Y.H.); frost_hyx@163.com (Y.H.); grandy@chd.edu.cn (W.L.); peilili@chd.edu.cn (L.P.)

**Keywords:** skid resistance, 3D macrotexture characteristics, feature importance analysis, friction coefficient, Bayesian-LightGBM

## Abstract

The lack of skid resistance performance is a crucial factor leading to road traffic accidents. The pavement surface friction is an essential indicator for measuring the skid resistance. The surface texture structure significantly affects the friction between the tire and the pavement, determining the pavement skid resistance. To deeply study the relationship between surface texture structure and pavement skid resistance performance, two types of asphalt mixture specimens, asphalt concrete (AC) and open-graded friction course (OGFC), are prepared for the skid resistance performance test. Firstly, a high-precision 3D smart sensor Gocator 3110 is used to collect the 3D point cloud data of the asphalt mixture surface texture. The British pendulum tester is used to measure the friction. Secondly, ten feature parameters are extracted to describe the 3D macrotexture characteristics. A data set containing 10 features and 200 groups of texture and friction data was also constructed. Meanwhile, the influence of macro-texture features on the skid resistance performance is discussed. Finally, an optimized Bayesian-LightGBM model is trained based on the constructed dataset. Compared with LightGBM, XGBoost, RF, and SVR algorithms, the Bayesian-LightGBM model can evaluate skid resistance more accurately. The R^2^ value of the proposed model is 92.83%. The research results prove that ten macrotexture features contribute to the evaluation of skid resistance to varying degrees. Furthermore, compared with AC mixture specimen, the texture surface of OGFC mixture specimen has more obvious height characteristics and higher roughness. The skid resistance of OGFC mixture specimens is better than that of AC.

## 1. Introduction

**Figure 1:**
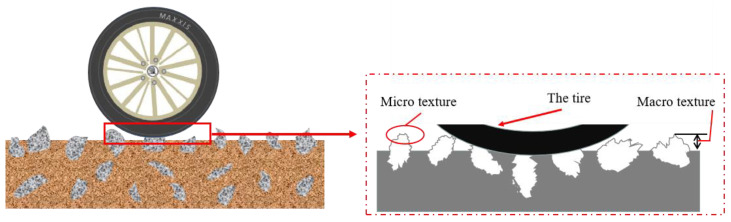
The schematic diagram of tire contact with road surface.

The lack of skid resistance of the road surface will seriously affect the road safety performance. The skid resistance depends on the friction between the vehicle tires and the road surface. The lack of friction will lead to the vehicle not being able to brake in time, thus causing traffic accidents [1]. Theory and engineering practice show that surface texture properties have a particularly significant effect on friction and directly determine skid resistance [2]. The roughness of the texture affects the friction generated by the contact between the tire and the road surface, as shown in Figure 1. Asphalt pavement texture can be divided into four categories: micro-texture, macro-texture, mega-texture, and unevenness. In the 18th world road meeting table committee, micro-texture and macro-texture will affect the skid resistance performance to varying degrees [3].

Many scholars have researched the relationship between road surface texture structure and skid resistance performance [4,5,6]. The related theories, design ideas, technical methods proposed have far-reaching effects on the research of the skid resistance mechanism of asphalt pavement and the reduction in traffic accidents, and also proved that the surface texture significantly affects the skid resistance of the pavement. Some studies have established the relationship between mean profile depth (MPD) or mean texture depth (MTD) and surface friction and tried to analyze the trend of friction coefficients by these factors [7,8,9,10]. However, limited results have been achieved, mainly because MPD and MTD only characterize a few pavement texture properties [11]. Therefore, more texture factors should be explored to illustrate the 3D texture information of pavements. The emergence of 3D laser acquisition equipment and the development of 3D image acquisition technology provides advantages for studying 3D texture information of pavements [12]. Wei et al. [13] used a three-dimensional laser to measure the relevant parameters (MTD, Ra, Rsk, Rku, and fractal dimension) of the surface morphology and analyzed the effect of these parameters on the skid resistance performance. Du et al. [14] collected pavement texture data with a 3D laser camera. They extracted 27 parameters from 3D texture data, including angle parameters, contour parameters, height parameters, functional parameters, and volume parameters. These parameters are used to verify the validity of the skid resistance evaluation model based on the texture of the contact area. Ji et al. [15] used 3D laser equipment to collect the 3D data of pavement texture and extracted macro-texture parameters (Ra, Rq, and Rz). The experimental results showed that a stable correlation was maintained between these parameters and the skid resistance performance. Han et al. [16] significantly improved the accuracy of the prediction model of pavement friction coefficient by analyzing the pavement skid resistance considering the micro-texture features of the pavement surface. Wang et al. [17] used binocular reconstruction method to obtain 3D texture data of the pavement surface and analyzed the relationship between 3D texture features and friction coefficient. It is proved that the aggregate type and 3D texture can directly evaluate the skid resistance performance of pavement. Kováč et al. [18] used a laser scanner to measure the pavement texture and calculated 85 different 3D texture parameters. It was found that using only a single texture parameter to determine the friction is not sufficient. Hu et al. [19] used a handheld 3D laser scanner to obtain pavement texture information. Through the 3D reconstruction of pavement texture images, eight texture parameters (Sa, Ssk, Sku, Sq, Sdq, Sdr, Spd, and Spc) reflecting pavement texture information from various angles were extracted and characterized, and a linear regression-based friction coefficient prediction model was constructed. The above research shows that the macro-texture of the pavement has a significant impact on the skid resistance performance. However, most studies reflect the relationship between texture features and skid resistance by means of correlation coefficient calculation. The correlation coefficient measures only the linear relationship between variables and is not sensitive to nonlinear relationships. When dealing with multidimensional data, the method has limited accuracy. Therefore, this paper constructs a machine learning model to automatically calculate the importance of 3D texture features to study the impact of macro-texture on skid resistance performance.

Based on obtaining a large amount of texture information, some studies have used conventional statistics and numerical analysis methods to construct skid resistance performance evaluation models. Al-Assi et al. [20] used the close-range photogrammetry (CRP) technique to measure micro-texture information, calculate micro-texture parameters from the acquired and analyzed pavement surface images, and input the texture parameters into the Persson model to predict the friction coefficient. Yang et al. [21] used wavelet transform to map texture depth features to different wavelength regions, and then used cross-validation and stepwise multiple linear regression methods to correlate texture features and friction coefficients. Athiappan et al. [22] developed a model for predicting skid resistance based on texture depth using a regression algorithm. It was found that texture depth has a greater effect on skid resistance. The model can be used to evaluate the variation of skid resistance of urban roads. Pattanaik et al. [23] developed a British pendulum number (BPN)-based skid resistance evaluation model using experimental observations to model the coefficient of friction using a group method of data handling (GMDH) and multi-gene symbolic genetic programming (MSGP). The proposed model can simplify extreme nonlinear deviations in data and predict friction performance. Heriberto et al. [24] established a prediction model of long and short-term skid resistance based on multiple linear regression combining pavement structure data, annual average daily traffic volume data, and lane number. Meanwhile, the covariance analysis method was introduced to analyze the main factors affecting skid resistance performance. Galvis et al. [25] used Markov chains to establish a network-level attenuation model of skid resistance performance. To prove the applicability of the proposed method, a numerical case study was carried out using a sample of 564-lane highway sections in Texas. The results showed that the Markov chain could effectively simulate the attenuation process of pavement skid resistance performance. Qda et al. [26] conducted multi-scale power spectrum analysis on 3D surface texture and constructed a multivariate linear fitting model to predict asphalt pavement friction. Goulias et al. [27] built a structural equation model to analyze the influence of pavement materials on skid resistance. They used statistical methods to predict the pavement friction coefficient. Chen et al. [28] established a pavement friction prediction model based on macro-texture and micro-texture by using multiple nonlinear regression analysis.

Although the above studies have constructed many parameters to describe texture information, they used relatively simple structures algorithms to analyze the effect of texture parameters on skid resistance performance. The methods proposed in some studies only reflect the linear relationship between a single texture feature and the skid resistance performance, and the utilization rate of the original data is low.

With the rapid advancement of artificial intelligence, increasing scholars have been applying it to related research in road engineering [29,30,31,32]. Pei et al. [33] proposed an aggregate shape classification model based on extreme gradient boosting (XGBoost). The results show that the XGBoost classification model can effectively use aggregate 2D images to classify aggregate shapes. They were aiming at the characteristics of poor continuity and low contrast of pavement cracks. Najafi et al. [34] proposed a vehicle collision rate prediction model based on an artificial neural network (ANN) using surface friction, traffic level, and speed limit features. The model proposed in the article can accurately predict the incidence of accidents. The results can be used as an indicator to determine the priority of safety improvement projects to improve the safety of highway networks. Yu et al. [35] discussed the influence mechanism of speed and pavement texture on the friction coefficient of the pavement and used the BP neural network method to construct the prediction model of the friction coefficient of the asphalt pavement. Zhan et al. [36] evaluated the influence of aggregate characteristics and texture features on skid resistance performance and used random forest algorithm to build a friction coefficient prediction model. Chen et al. [37] used a deep learning network to recognize road texture. The GAN model was used to enhance the original texture image data set. They used Densenet networks to classify raw data sets. It was found that the performance of deep learning network for pavement texture recognition is better than that of traditional methods.

The successful application of machine learning theory in road engineering has brought new prospects for evaluating pavement skid resistance performance. Therefore, the purpose of this paper is to explore the application of intelligent learning algorithms in pavement skid resistance assessment. Light gradient boosting machine (LightGBM) is an improved boosting framework algorithm released by Microsoft in 2017 with decision trees as the base learner, which overcomes the shortcomings of XGBoost that consumes a lot of time and space [38,39]. The LightGBM algorithm supports distributed learning, can quickly process massive amounts of data, and can be used for various sorting, classification, and regression problems in machine learning. In summary, the main contributions of this paper are as follows:

(1) In this paper, 200 sets of surface texture and friction data of different gradation types are collected. Meanwhile, 10 macro-texture parameters are extracted from the 3D texture data.
(2) To overcome the drawbacks of poor nonlinear fitting performance and the limited accuracy of traditional methods, a LightGBM-based model for correlation analysis between 3D texture features and skid resistance is constructed, and the importance of 3D texture features is obtained.
(3) A friction coefficient prediction model based on Bayesian-LightGBM is constructed according to the macro-texture features. A Bayesian optimization algorithm is used to improve the predictive performance of the model. The research method proposed in this paper effectively improves the evaluation accuracy of skid resistance performance based on 3D texture feature parameters. It is expected to promote the improvement of road intelligent detection technology.

## 2. Data and Method

### 2.1. Preparation of Asphalt Mixture Specimen

The skid resistance performance of asphalt mixture specimens of different aggregate and gradation types are different. In this study, two types of asphalt mixture specimens, AC and OGFC, are prepared for indoor skid resistance evaluation experiments. These specimens are prepared from limestone and common asphalt. To enrich the surface texture of the mixture specimens, refer to the specification for preparing the mixture gradation type and quantity, as shown in Table 1.

**Figure 2:**
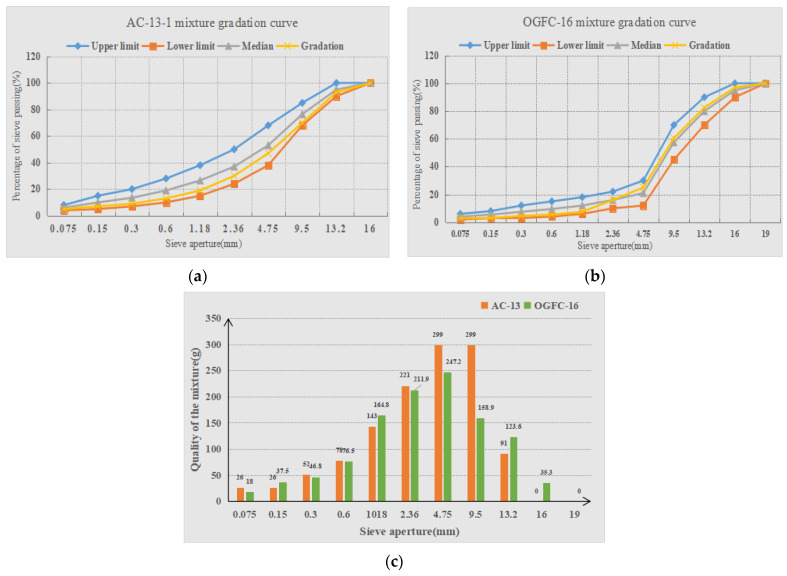
The gradation curves and the quality of the mixture. (**a**) AC-13-1 gradation curve. (**b**) OGFC-16 gradation curve. (**c**) Quality of the mixture.

Taking AC-13 and OGFC-16 in Table 1 as examples, the gradation curves of the mixture specimens and quality of the mixture are shown in Figure 2.

The preparation of asphalt mixture specimens of different gradation types mainly includes the following steps: (1) The aggregates are sieved into different sizes according to the standard sieve. (2) The actual amounts of mineral powder and aggregates are weighed according to the gradation information in Figure 2. (3) Asphalt mixture specimens of size 30 cm×30 cm×5 cm are prepared according to road design specifications.

### 2.2. Collection of 3D Texture Data 

**Figure 3:**
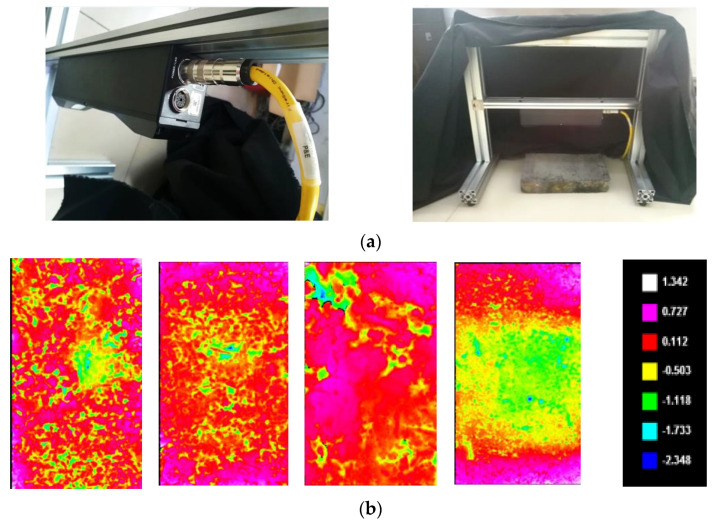
Surface texture 3D point cloud data collection. (**a**) 3D data acquisition equipment construction. (**b**) Pseudo color map of texture depth data.

In this paper, the 3D intelligent sensor Gocator 3110 (produced by LMI technologies Inc. Burnaby, BC, Canada)is used to scan the surface texture of the specimen to collect 3D texture point cloud data. The construction and scanning process of the 3D point cloud data acquisition system for the surface texture of asphalt mixture specimens based on Gocator 3110 is shown in Figure 3a. The specimen is placed in the scanning field of the Gocator 3110. The 3D characteristics of the surface texture of the asphalt mixture sample are scanned according to the instrument specifications. The 3D texture point cloud data will be exported and saved into *. CSV format files. Figure 3b shows the pseudo color map of the 3D texture depth data collected by Gocator 3110. Different colors indicate different texture depths. It can be seen that the surface texture structure is significantly different in each image.

### 2.3. Skid Resistance Test

**Figure 4:**
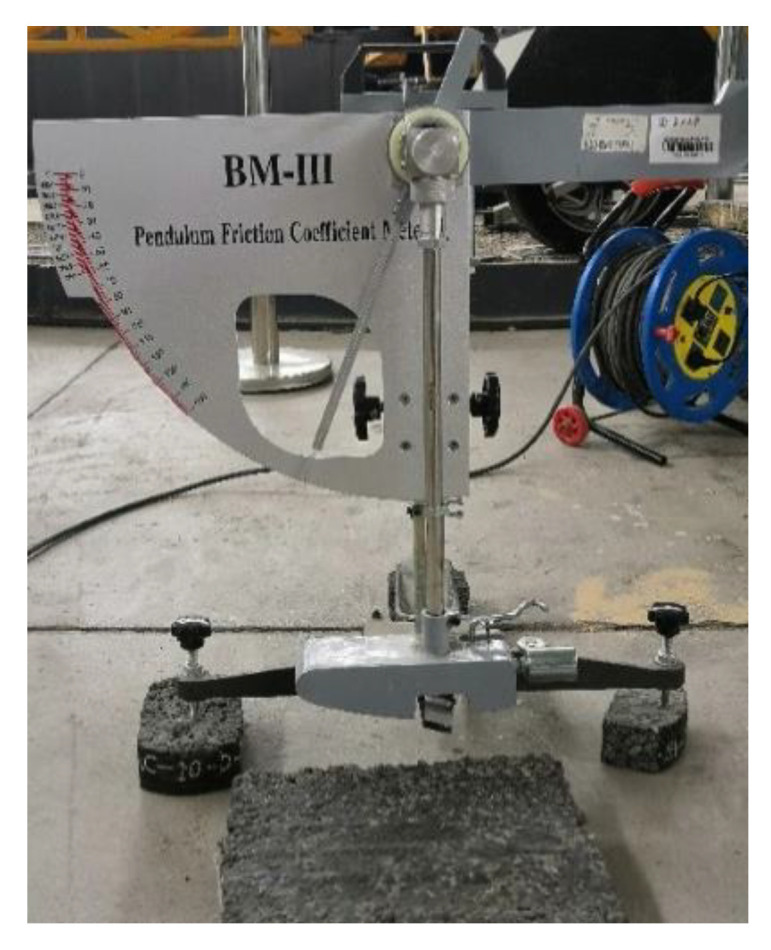
British Pendulum Tester.

In this study, the friction coefficient of the specimen surface is measured by the British pendulum tester (BPT), as shown in Figure 4. The principle of the BPT is to use the pendulum arm in the process of swinging the pendulum and the contact surface friction work, resulting in the loss of gravitational potential energy of the pendulum, according to the principle of energy conservation friction coefficient. According to the operating specifications and steps of the BPT [40], the friction coefficient of different asphalt mixture specimens is measured.

### 2.4. Preprocessing 3D texture Data 

#### 2.4.1. Repair of Missing Values in 3D Texture Point Cloud Data

**Figure 5:**
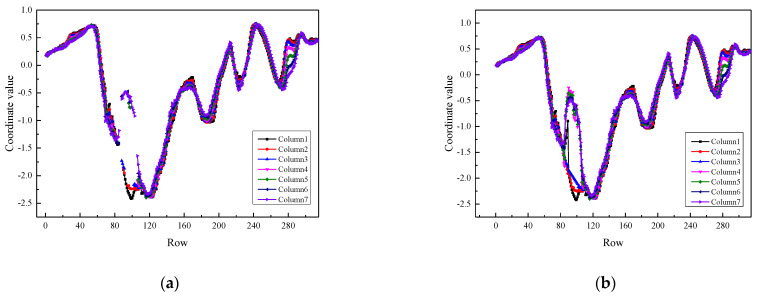
Missing value filling results based on third-order B-spline interpolation method. (**a**) Missing values; (**b**) Repair results of missing values.

To accurately calculate the 3D texture parameters, missing values in the raw data need to be filled in. The original data missing type is random missing, and there are not many consecutive missing values in the original data. The data distribution feature is more suitable for interpolation method to solve missing values. The interpolation function curve of B-spline interpolation method has good smoothness, so the third-order B-spline interpolation method is used to fill the missing values in 3D point cloud data. Firstly, according to the known point cloud data, the unknown points are obtained by inverse algorithm to determine the B-spline curve, and the point cloud value is calculated according to the position of missing data. Figure 5 shows the missing values fitting results. Different colors represent point cloud data distribution curves of different columns. The more the interpolated data match the trend of the original data, the better the interpolation result. Figure 5 shows that b-spline interpolation method can effectively restore the original distribution state of data.

#### 2.4.2. Feature Extraction of 3D Macrotexture 

**Figure 6:**
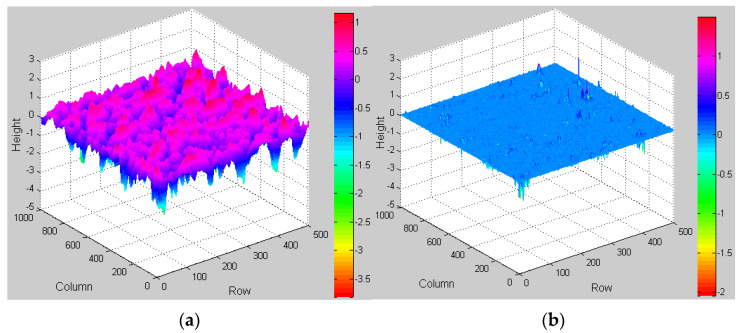
The texture separation results are based on Gaussian filtering. (**a**) Macro-texture. (**b**) Micro-texture.

The original surface texture of the asphalt mixture specimen is divided into micro-texture and macro-texture. To prepare the quantitative characterization of the macro-texture index, it is necessary to separate the macro-texture and micro-texture. Gaussian filtering algorithm is used to extract macro texture data from the original texture data. The sampling points of the original 3D texture data in the row and column directions are 500 and 1000, respectively. The sampling interval is Δ=0.1 mm. The cutoff wavelength of the Gaussian filter is set to $\lambda_{c}=0.5\;\mathrm{mm}$ according to the boundary of wavelength differentiation between macro-texture and micro-texture. The half-window width of the Gaussian filtering power function is $m=\lambda_{c}/\Delta =5\;\mathrm{mm}$ [41]. The results of the texture separation algorithm based on Gaussian filtering in MATLAB (From MathWorks company, Natick, MA, USA) are shown in Figure 6. The red curve represents the extracted macro profile. It can be seen from Figure 6 that the extracted macrotexture effectively retains the information of the original texture.

To effectively describe the 3D texture information of asphalt pavements, ten macrotexture features are calculated and extracted to evaluate the skid resistance based on MATLAB [13,17,42,43], as shown in Table 2.

1. Correlated characterization indexes of height

To examine the height features of the pavement texture, the three indicators of the arithmetic mean deviation of the profile Ra, the root mean square deviation of the profile Rq, and MTD are chosen as height-associated indicators in this work. The specific definition is as follows:

(1) $$Ra=\frac{1}{M\times N}\sum_{i=1}^{N}\sum_{j=1}^{M}\left|z(x_{i},y_{j})\right|$$

(2) $$Rq=\sqrt{\frac{1}{M\times N}\sum_{i=1}^{N}\sum_{j=1}^{M}{\left[z(x_{i},y_{j})\right]}^{2}}$$

(3) $$MTD=\frac{1}{M\times N}\sum_{i=1}^{N}\sum_{j=1}^{M}\left[z_{p}(x,y)-z(x_{i},y_{j})\right]$$

where z(x,y) represents the height characteristics of the textured topography based on the baseline. M and N are the width and the length of the sampling range, respectively. $z_{p}(x,y)$ represents the line of profile peaks in the sampling range.

2 Correlated characterization indexes of wavelength

In addition to the distribution in the height direction, the distribution characteristics of the pavement texture in the horizontal direction also affect the skid resistance performance. The characterization indicators related to the wavelength include: mean distance of profile unevenness Sm, mean distance between single profile peaks S, root mean square wavelength of the profile Wq, and surface roughness area ratio R [44].

(4) $$Sm=\frac{1}{p}\sum_{i=1}^{n}Sm_{i}$$

where p is the number of irregular spacing, and $Sm_{i}$ represents the distance of the $i^{th}$ microscopic irregularity.

(5) $$S=\frac{1}{p}\sum_{j=1}^{m}S_{j}$$

where $S_{j}$ represents the horizontal distance between the $j^{th}$ adjacent peak.

The profile root means square wavelength Wq is 2π times the ratio of Rq to profile root mean square slope Sq, and its definition is shown in Equations (6) and (7). It measures the average distance between the peaks (or valleys) of the texture profile in the sampling range.

(6) $$Wq=2\pi \frac{Rq}{Sq}$$

(7) $$Sq\;=\sqrt{\frac{1}{\left(M-1\right)\times \left(N-1\right)}\sum_{i=1}^{N-1}\sum_{j=1}^{M-1}{\left[\left(\frac{z(x_{i+1},y_{j})-z(x_{i},y_{j})}{\Delta x}\right)+\left(\frac{z(x_{i},y_{j+1})-z(x_{i},y_{j})}{\Delta y}\right)\right]}^{2}}$$

R is expressed by the ratio of the actual area $A_{1}$ of the surface texture to its nominal area $A_{2}$ (that is, the projected area of the surface texture on the horizontal plane). The larger the value of this index, the rougher the textured surface. It is defined in the Equations (8) and (9).

(8) $$R=\frac{A_{1}}{A_{2}}$$

(9) $$A_{1}\;=(\Delta x\Delta y)\sum_{i=1}^{N-1}\sum_{j=1}^{M-1}\sqrt{\left[{\left(\frac{z(x_{i+1},y_{j})-z(x_{i},y_{j})}{\Delta x}\right)}^{2}+{\left(\frac{z(x_{i},y_{j+1})-z(x_{i},y_{j})}{\Delta y}\right)}^{2}\right]+1}$$

3 Correlated characterization indexes of shape

Due to the different shapes and sharpness of the profile curved surface, the surface texture also shows different skid resistance performance. Root mean square slope of profile Sq, profile skewness Rsk, and profile steepness Rku reflect the distribution changes of the profile curved surface. Rsk and Rku are calculated as follows:

(10) $$Rsk=\frac{1}{Rq^{3}}\frac{1}{M\times N}\sum_{i=1}^{N}\sum_{j=1}^{M}z^{3}(x_{i},y_{j})$$

(11) $$Rku=\frac{1}{Rq^{4}}\frac{1}{M\times N}\sum_{i=1}^{N}\sum_{j=1}^{M}z^{4}(x_{i},y_{j})$$

## 3. Model Construction Method

### 3.1. Light Gradient Boosting Machine

Conventional statistics is a numerical analysis, including a single factor and the friction coefficient, and is a linear regression equation description. Machine learning algorithms can comprehensively consider multiple feature factors to predict the friction coefficient value. The LightGBM algorithm has more substantial stability, generalization ability, and higher calculation efficiency, compared with the traditional regression prediction algorithm. It can handle multi-factor regression prediction problems accurately. The principle of LightGBM is similar to XGBoost, and the residuals are approximated by the first-order and second-order Taylor expansions of the loss function. The algorithm builds multiple regression trees to form a tree group and makes the predicted value of the tree group close to the true value to improve the prediction accuracy of the model. When constructing the $t^{th}$ tree in the model [45]:

(12) $${\hat{y}}_{i}^{\left(t\right)}=\sum_{k=1}^{t}(f_{k}(x_{i}))={\hat{y}}_{i}^{\left(t-1\right)}+f_{t}(x_{i})$$

$f_{k}$ represents the $k^{th}$ tree, $f_{k}(x_{i})$ is the output score of the $k^{th}$ tree for the input $x_{i}$, and ${\hat{y}}_{i}^{\left(t\right)}$ is the prediction result of the sample $x_{i}$ of the combined t-tree model. Every time a new regression tree is constructed, the loss function of the model will change. The training error and regular term of the *t* ∑ 1 tree in front of the tree are known terms, so the objective function is:

(13) $$Obj^{\left(t\right)}=\sum_{i=1}^{n}l(y_{i},{\hat{y}}_{i}^{\left(t-1\right)}+f_{t}(x_{i}))+\Omega (f_{t})+cons\tan t$$

If the mean square error is used as the loss function, and the objective loss function can be rewritten as:

(14) $$\begin{array}{l} Obj^{\left(t\right)}=\sum_{i=1}^{n}{\left(y_{i}-({\hat{y}}_{i}^{\left(t-1\right)}+f_{t}(x_{i}))\right)}^{2}+\Omega (f_{t})+cons\tan t\\ =\sum_{i=1}^{n}\left[2({\hat{y}}_{i}^{\left(t-1\right)}-y_{i})f_{t}(x_{i})+f_{t}{\left(x_{i}\right)}^{2}\right]+\Omega (f_{t})+cons\tan t \end{array}$$

For a general loss function, XGBoost performs a second-order Taylor approximation expansion of the error function, and its loss function is expressed as Equation (15).

(15) $$\sum_{i=1}^{n}l(y_{i},{\hat{y}}_{i}^{\left(k-1\right)}+f_{k}(x_{i}))=\sum_{i=1}^{n}[l(y_{i},{\hat{y}}_{i}^{\left(k-1\right)})+l^{\prime }(y_{i},{\hat{y}}_{i}^{\left(k-1\right)})f_{k}(x_{i})+\frac{1}{2}l^{\prime \prime }(y_{i},{\hat{y}}_{i}^{\left(k-1\right)})f_{k}^{2}(x_{i})]$$

$g_{i}$ represents the first derivative of the loss function of the $i^{th}$ sample and $h_{i}$ is the second derivative of the loss function of the $i^{th}$ sample.

(16) $$g_{i}=l^{\prime }(y_{i},{\hat{y}}_{i}^{\left(k-1\right)})$$

(17) $$h_{i}=l^{\prime \prime }(y_{i},{\hat{y}}_{i}^{\left(k-1\right)})$$

Therefore, a new objective function is obtained:

(18) $$Obj\approx \sum_{i=1}^{n}\left[l(y_{i},{\hat{y}}_{i}^{\left(k-1\right)})+g_{i}f_{k}(x_{i})+\frac{1}{2}h_{i}f_{k}^{2}(x_{i})\right]+\Omega (f_{k})$$

**Figure 7:**
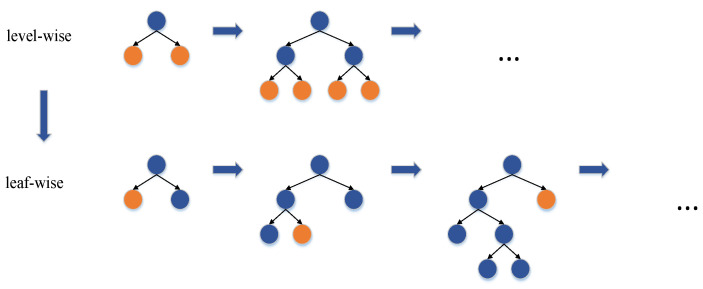
Level-wise and leaf-wise.

It can be seen that the target loss function is composed of training errors and regular terms. The key to the tree model is to build a decision tree that minimizes the objective function. The essential step in this process is the splitting strategy of the leaf nodes. The split methods of the two algorithms are shown in Figure 7. XGBoost uses a level-wise splitting strategy. This split method splits all nodes at each level of the tree, regardless of the split gain of the node. It increases the calculation time of the model to a certain extent. The leaf-wise split strategy in LightGBM selects the node with the largest split gain among the current leaf nodes for splitting while limiting the maximum depth of the tree, effectively avoiding the overfitting problem of the model and controlling the complexity of the tree.

**Figure 8:**
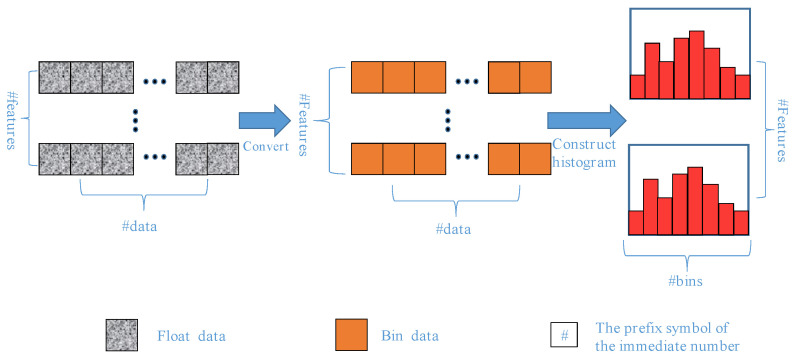
Sort structure based on histogram optimization.

The sorting method based on histogram optimization is also the advantage of the LightGBM. Figure 8 shows the process of the histogram sorting method. This method divides continuous values into discrete domains, that is, data binning. Taking floating-point data as an example, the constant values in an interval will be regarded as a discrete domain. Then, the features are sorted according to the histogram with these discrete domains as precision units.

In summary, XGBoost and LightGBM can solve the problem of multiple regression prediction and can also be used to analyze the importance of features.

### 3.2. Bayesian-LightGBM Fusion Model Construction

**Figure 9:**
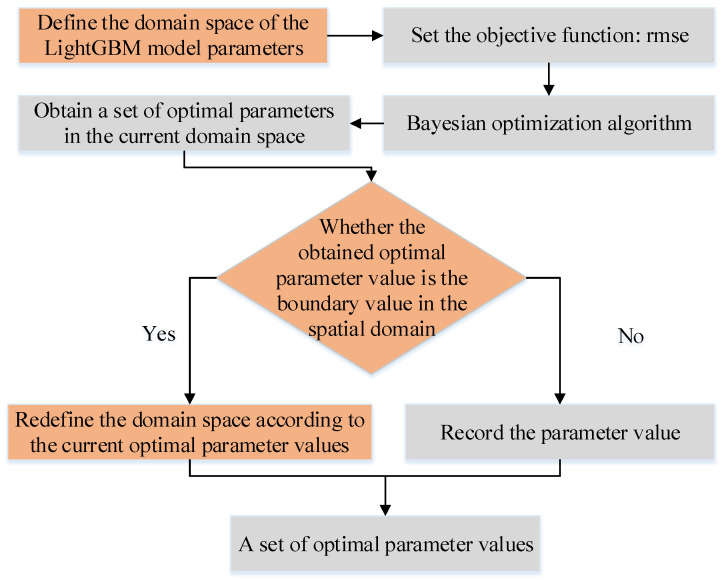
Bayesian optimization algorithm parameter iteration process.

To improve model performance, Bayesian optimization and LightGBM fusion method are proposed in this paper. Standard parameter optimization methods include grid search (GS), random search (RS), genetic algorithm (GA), particle swarm optimization algorithm (PSO), and Bayesian optimization algorithm. Although GS and RS algorithms are easy to implement, they easily fall into local optimal problems; GA and PSO can be classified as a group optimization algorithm. This type of algorithm has high complexity and requires enough original sample data, which increases the amount of calculation to a certain extent and reduces the efficiency of searching for the optimal solution. Therefore, to further improve the model performance and more accurately fit the observed and predicted values of the friction coefficient, this study constructs a friction coefficient prediction model based on the Bayesian-LightGBM fusion algorithm. The Bayesian optimization algorithm is mainly used to optimize the essential parameters of the LightGBM, as shown in Table 3 Bayesian optimization algorithm uses the evaluation results of the previous objective function to establish a new substitution function (probability model) to find the value that minimizes the objective function. The Bayesian optimization method is different from other methods. It refers to the evaluation result of the previous objective function when looking for the next set of hyper-parameters, thus improving the efficiency of parameter optimization. Figure 9 is the process of the Bayesian optimization algorithm seeking the optimal solution of LightGBM parameters. The LightGBM model parameters are first initialized. Secondly, the domain space is defined, that is, the boundary value of the parameter. Then, the Bayesian optimization algorithm performs parameter search and optimization in the domain space to obtain the optimal parameter combinations in Table 3. Finally, the Bayesian-LightGBM friction coefficient prediction model is constructed based on the optimal parameter combinations.

### 3.3. Model Evaluation

The indicators in Table 4 are selected to quantitatively evaluate the prediction model’s performance before and after optimization. $R^{2}$ represents the goodness of fit, the closer the value is to 1, the better the model fits the observed value and the predicted value; root means square error (RMSE) reflects the deviation between the observed value and the predicted value. The smaller the value, the better the model’s performance; mean absolute percentage error (MAPE) indicates the average deviation degree of the predicted result from the observed result. The error of each point is normalized.

## 4. Feature Selection and Friction Coefficient Prediction Results

### 4.1. Feature Selection Based on LightGBM

**Figure 10:**
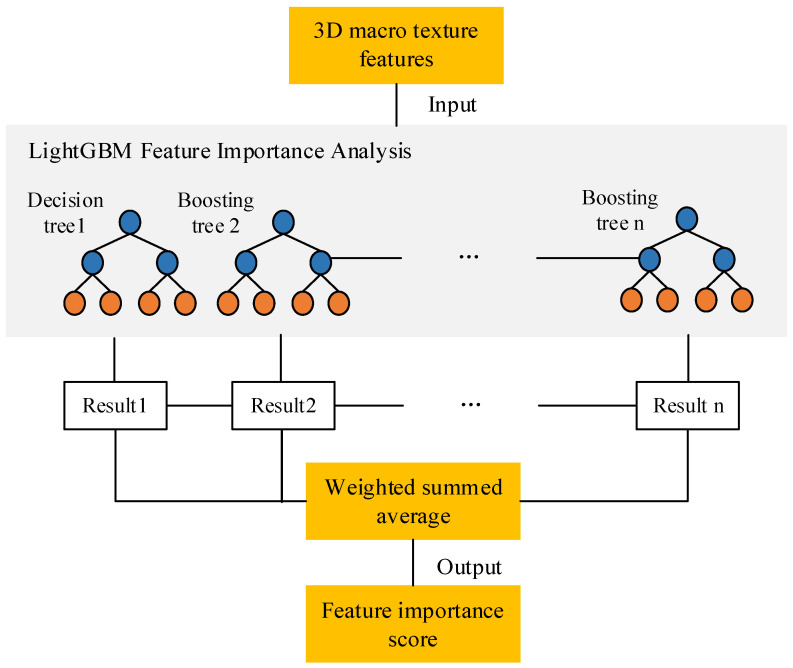
The analysis process of feature importance based on LightGBM.

To clarify the mechanism of macrotexture features affecting skid resistance performance, it is necessary to analyze the correlation between them. The traditional correlation coefficient analysis method can only reflect the linear relationship between features and is not sensitive to nonlinear relationships. When the original data dimension is large, this method has a large error. Therefore, this paper investigates the correlation between texture features and skid resistance based on the LightGBM model. Figure 10 shows the analysis process of feature importance based on LightGBM. All the 3D macrotexture features are input into the LightGBM model, and then the model determines the importance of features based on their contribution in the process of constructing the boosting tree. The more times a feature is selected to build a boosting tree, the higher its feature importance score will be.

**Figure 11:**
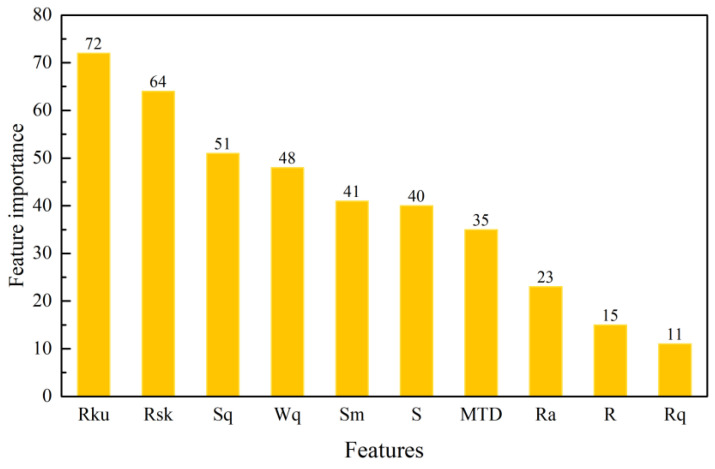
Feature importance analysis results.

Figure 11 shows the ranking results of feature importance based on LightGBM. According to the results of feature importance analysis, it can be seen that each 3D macrotexture feature has a different degree of influence on the skid resistance performance. The 3D feature parameters Rsk, Rku, and Sq have higher feature importance score, and the correlation between these parameters and the friction coefficient is highly significant, followed by Wq, Sm, S, MTD and Ra. R and Rq have lower feature importance scores, indicating that these features have the weakest correlation with the friction coefficient.

### 4.2. Prediction Results of Friction Coefficient Based on Bayesian-LightGBM Model

**Figure 12:**
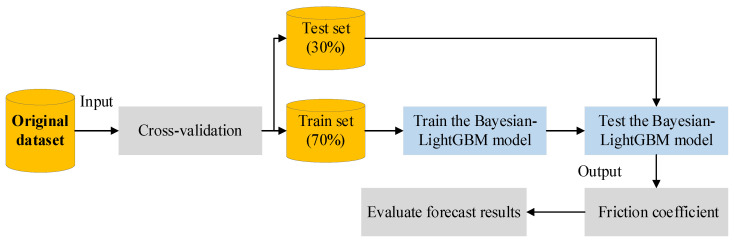
Friction coefficient prediction model based on LightGBM algorithm.

In this study, a friction coefficient value prediction model under multi-feature conditions is constructed based on the LightGBM algorithm. Figure 12 shows the construction process of the friction coefficient prediction model. A total of 200 sets of surface texture and friction data are collected. The original data set is divided into training and test sets according to the ratio of 7:3. Then, a prediction model of friction coefficient based on Bayesian-LightGBM is constructed using 3D macrotexture features. The Bayesian optimization algorithm is used to search the optimal value of LightGBM model parameters to improve the prediction accuracy. The parameter optimization results are shown in Table 5. Parameter optimization can avoid model overfitting and reduce model complexity.

**Figure 13:**
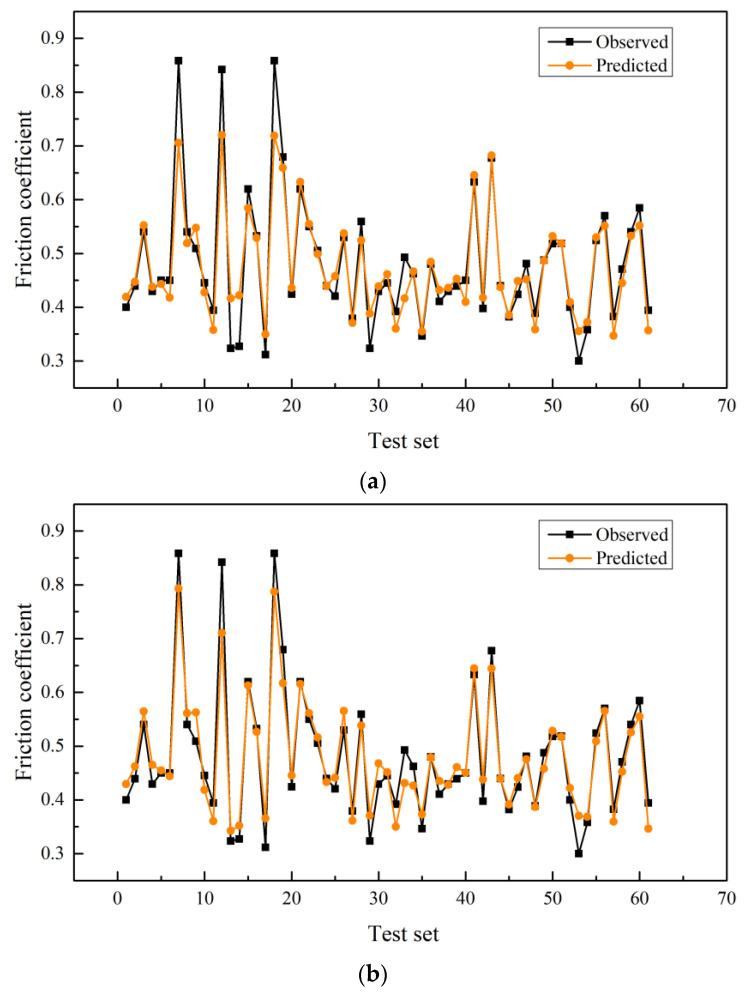

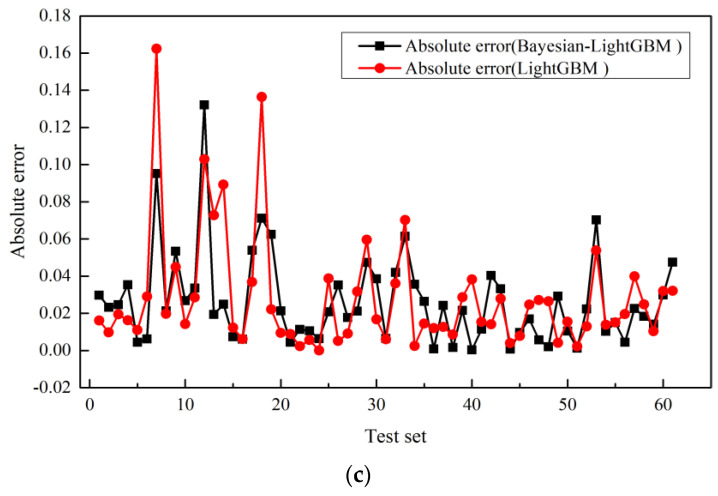
Prediction results of friction coefficient. (**a**) LightGBM model prediction results. (**b**) Bayesian-LightGBM model prediction results (**c**) Distribution chart of the absolute error between the observed value and the predicted value.

Figure 13a shows the comparison between the prediction curve of the original LightGBM model and the real curve. Figure 13b shows the comparison between the prediction curve of the original Bayesian-LightGBM model and the real curve. Figure 13c shows the absolute error comparison of the model before and after optimization. The analysis of Figure 13a,b shows that the predicted curves of the original LightGBM model are generally consistent with the distribution of the true curves. However, when the friction coefficient is large, the prediction error of the original LightGBM model is large. The predicted value based on Bayesian-LightGBM model is closer to the real value, and the prediction accuracy is improved when the friction coefficient is large. The distribution of error curves effectively verifies the above conclusions. At the same time, the experimental results prove that parameter optimization can effectively improve the accuracy of the model.

## 5. Results Discussion

### 5.1. Friction Coefficient and 3D Texture Features 

**Figure 14:**
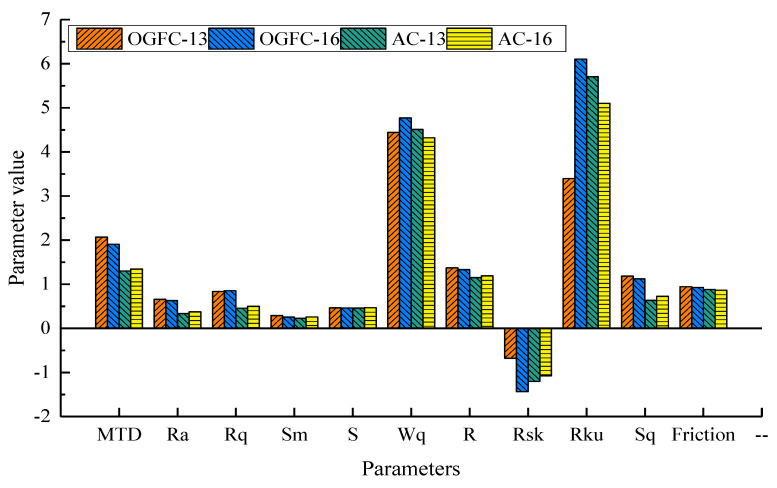
The histograms of the data distribution of macrotexture parameters and friction coefficients.

Friction data for four different gradation types (OGFC-13, OGFC-16, AC-13, and AC-16) are analyzed to investigate the effect of 3D texture features on the skid resistance performance. Figure 14 shows the histograms of the data distribution of macrotexture parameters and friction coefficients for the four asphalt mixture specimens. Figure 14 shows that the friction coefficient of OGFC-13 is greater than that of OGFC-16, and the friction coefficient of AC-13 is greater than that of AC-16. Meanwhile, the friction coefficient of OGFC gradation type is greater than that of AC gradation type. This result shows that the skid resistance of the OGFC gradation type is better than that of the AC gradation type.

MTD, Ra, and Rq describe the details of the height direction of 3D macrotexture. By analyzing the data distribution of these parameters, the height characteristics of OGFC gradation type are more significant than that of AC gradation type. Sm, S, Wq, and R describe the details of the wavelength direction of 3D macrotexture. There is no significant difference in the distribution of micro unevenness spacing and single peak spacing of texture profile among the four gradation types. A higher R indicates a rougher surface. As a result, the surface texture of the specimens of the OGFC-13 and OGFC-16 gradation types is rougher than that of AC-13 and AC-16. Rsk, Rku, and Sq describe the geometric shapes of the surface texture structure. Rsk is less than 0 for all four gradation types, indicating that the surface texture profile of these specimens has a “sharp valley, smooth peak” structure. Rku is more than 3 for all four gradation types, indicating that the amplitude distribution of the texture profile of these specimens is steep. Sq reflects that, compared with AC-13 and AC-16, OGFC-13 and OGFC-16 have a more obvious average incline of their curved profiles. Comprehensive analysis of the above 3D texture structure and gradation information shows that the specimens of OGFC gradation type have more coarse aggregate content, deeper texture depth, and rougher texture surface. Enough texture depth is conducive to the braking of the vehicle and improves the skid resistance of the road surface to a certain extent. Therefore, the skid resistance of the specimens with OGFC gradation type is better than that of AC.

### 5.2. Analysis of the Predicted Friction Coefficient Results Based on Bayesian-LightGBM

**Figure 15:**
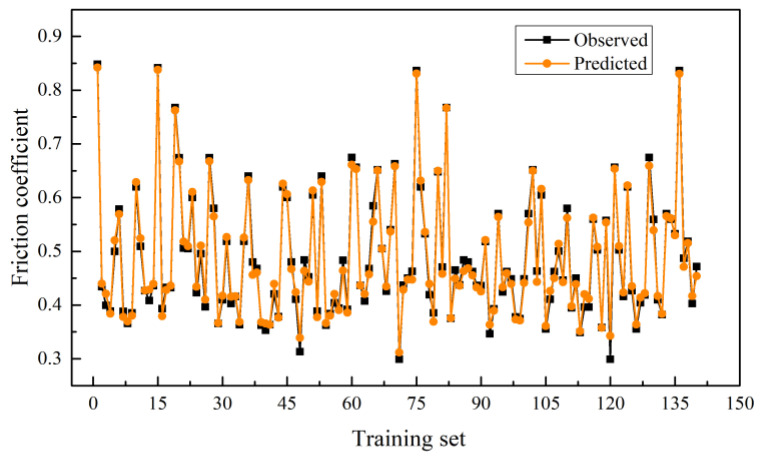
Prediction results of the training set based on Bayesian-LightGBM.

Figure 15 shows the prediction curve of training set and test set based on the Bayesian-LightGBM model. Table 6 shows the comparison results of the Bayesian-LightGBM model on the training set and the test set. The $R^{2}$ values of the model on the training and test sets are 98% and 93%, respectively. On the training set, the predicted and true curves match well. It can be seen that the model has good prediction ability on the training set and learns the pattern of the original data accurately. On the test set, the predicted values of the model are close to the true values. It shows that the model has strong generalization ability and can effectively generalize to data sets with the same distribution as the training set.

### 5.3. Comparison Analysis of Friction Coefficient Prediction Models

**Figure 16:**
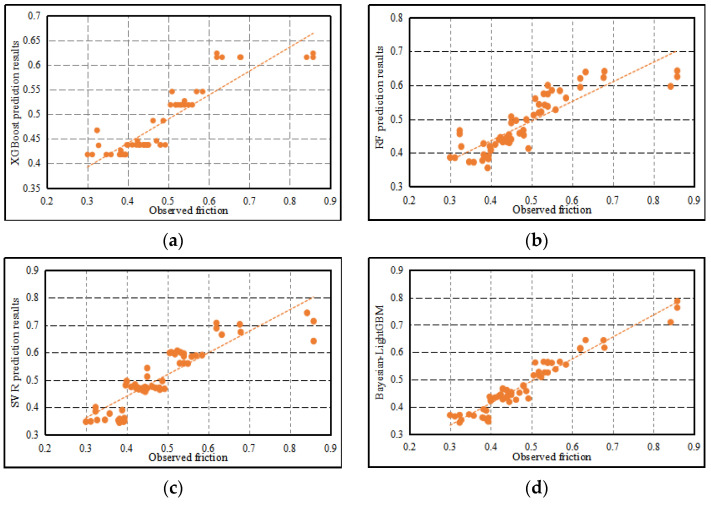
Compare the proposed model with other models. (**a**) The fitting result between the predictive value of the XGBoost and the observed value. (**b**) The fitting result between the predictive value of the RF and the observed value. (**c**) The fitting result between the predictive value of the SVR and the observed value. (**d**) The fitting result between the predictive value of the Bayesian-LightGBM and the observed value.

To prove the prediction accuracy of the proposed model, this paper compares it with XGBoost, random forest (RF), and support vector machine regression (SVR). The fitting effect comparison of the different models is shown in Figure 16. The evaluation results are shown in Table 7. The comprehensive analysis of Figure 16 and Table 7 shows that the Bayesian-LightGBM model has better nonlinear fitting performance under the condition of multi-dimensional data and can accurately evaluate the relationship between 3D macrotexture characteristics and skid resistance. The reasons are as follows: (1) We can see that the Bayesian-LightGBM model can better fit the observed values and predicted values. In particular, when the friction coefficient value is in the interval [0.7, 0.9], the predicted value of the optimized model is closer to the observed value, which is significantly improved compared with the previous predicted result. (2) Under the same data conditions, the distribution of predicted and true values of the Bayesian-LightGBM model is closer to the straight line of y=x. (3) Compared with RF, XGBoost, SVR, and LightGBM, the $R^{2}$ of the proposed Bayesian-LightGBM model is increased by 18%, 13%, 13.4%, and 3%, respectively, and the RMSE of the proposed Bayesian-LightGBM model decreases by 3.3%, 3.4%, 2.5%, and 0.6%, respectively.

## 6. Conclusions and Future Research

In this study, the effect of 3D macro-texture features on skid resistance is explored. Meanwhile, a Bayesian-LightGBM based evaluation model for skid resistance of asphalt pavement is constructed by using these features. After discussion and analysis, the following conclusions are drawn:

(1) The nonlinear relationship between 3D macrotexture features and skid resistance is analyzed based on LightGBM. The results of feature importance analysis show that each 3D macrotexture feature has a different degree of influence on the skid resistance performance. The 3D feature parameters Rsk, Rku, and Sq have a more significant effect on skid resistance performance, followed by Wq, Sm, S, MTD, and Ra. R and Rq have lower feature importance scores, indicating that these features have the weakest correlation with the friction coefficient.
(2) From the results of the feature importance analysis, we can conclude that the change of the pavement skid resistance performance results from the combined effect of different texture characteristic factors. A single index cannot reflect the influence mechanism of texture characteristics on skid resistance performance. More feature parameters should be selected to describe texture information, not limited to standard MTD features.
(3) The texture features and friction data of asphalt mixture specimens of different gradation types are analyzed. It is found that the specimens with OGFC-13 gradation type have the best skid resistance, followed by OGFC-16, AC-13, and AC-16. Compared with AC mixture specimen, the texture surface of OGFC mixture specimen has more obvious height characteristics and higher roughness. Therefore, the skid resistance of OGFC mixture specimens is better than that of AC.
(4) Bayesian-LightGBM is more suitable than traditional model to evaluate the effect of surface texture on skid resistance performance. By discussing the performance of the proposed model on the training set and test set, it is proved that the model has strong generalization ability and can effectively adapt to the data set with the same distribution as the original data.
(5) Compared with RF, XGBoost, SVR, and LightGBM, the $R^{2}$ of the proposed Bayesian-LightGBM model is increased by 18%, 13%, 13.4%, and 3%, respectively. The comparison results show that the Bayesian-LightGBM model can accurately fit the friction coefficient by using 3D macrotexture features. Bayesian optimization algorithm improves the nonlinear fitting ability of the LightGBM model under multi-dimensional condition.

Traditional feature analysis methods reflect a linear relationship between two variables and are insensitive to nonlinear relationships between multiple features. Therefore, the LightGBM algorithm is used to automatically calculate feature importance and measure the contribution of each feature to the evaluation of skid resistance. Furthermore, a friction coefficient prediction model based on Bayesian-LightGBM is constructed using these features. By analyzing the prediction results, it is found that the prediction effect of the optimized model is better than the original model in general. However, there is still some error in predicting the friction coefficient in the range of [0.8, 0.9]. The reason is that the amount of data distributed in the interval is low. Therefore, it is necessary to expand the dataset to support model training and improve the performance of the model in subsequent studies

The friction coefficient prediction model constructed in this paper provides the necessary theoretical methods and data support for efficient assessment of the skid resistance of asphalt pavements under massive data conditions. At the same time, the microstructure and water film thickness are also the key factors affecting skid resistance. Therefore, future research will mainly include three aspects: (1) We will expand the data set to help the model train better. (2) The different effects of macro-texture and micro-texture on skid resistance performance are discussed. (3) Factors, such as temperature, humidity, and water film, will be comprehensively considered to construct an evaluation model of skid resistance performance based on the fusion of intelligent optimization algorithms and machine learning models.

## Figures and Tables

**Table 1:** Asphalt mixture type.

Asphalt Mixture	Mixture Gradation Type	Species	Number
Asphalt Concrete (AC)	AC-20	3	1
	AC-16	3	1
	AC-13	3	1
	AC-10	3	1
	AC-5	3	1
Open Graded Friction Course (OGFC)	OGFC-16	3	1
	OGFC-13	3	1
	OGFC-10	3	1

**Table 2:** Definition of 3D texture feature parameters.

Parameters	Parameter Meaning
Arithmetic mean deviation Ra (mm)	Reflects the degree of dispersion of the profile amplitude relative to the baseline
Root mean square deviation Rq (mm)	Reflects the standard deviation of the amplitude distribution of the random surface texture profile
Mean texture depth MTD (mm)	Reflects the average depth of surface texture voids
Mean distance of profile unevenness Sm (mm)	Reflects the intersection density of profile and centerline
Root mean square wavelength Wq	Measures the average distance between the peaks (or valleys) of the texture profile in the sampling range
Surface roughness area ratio R	Reflects the unevenness of the texture surface relative to the median surface
Mean distance between single profile peaks S (mm)	Reflects the peak density of the profile
Skewness Rsk	Reflects the degree of asymmetry of the profile amplitude distribution deviating from the baseline
Steepness Rku	Reflects the sharpness of the profile surface distribution
Root mean square slope Sq	Reflects the root mean square of the slope of each point of the contour surface

**Table 3:** LightGBM model parameters.

Model Parameters	Parameter Adjustment Effect
max_depth	The maximum depth of the tree, when this value is large, the model is more complex and prone to overfitting
min_data_in_leaf	Minimum number of records for leaves, setting it to a larger value can prevent the tree from growing too deep
max_bin	Indicates the maximum number of features stored in the bin
num_leaves	It can be used to control the complexity of the tree
bagging_fraction	The proportion of data used in each iteration, used to speed up the training speed of the model
bagging_freq	Re-sampling is done every bagging_freq iterations, and the sampling ratio is bagging_fraction
feature_fraction	When the value is set to 0.8, it means that 80% of the features are randomly selected to construct a decision tree in each iteration
lambda_l1	Regular term L1
lambda_l2	Regular term L2

**Table 4:** Evaluation index.

Evaluation Index	Equation	Explanation
$R^{2}$	$R^{2}=1-\frac{\sum_{i=1}^{m}{\left(y_{i}-{\hat{y}}_{i}\right)}^{2}}{\sum_{i=1}^{m}{\left(y_{i}-y_{mean}\right)}^{2}}$	$y_{i}$ is the observed value; ${\hat{y}}_{i}$ is the predicted value; $y_{mean}$ represents the mean of the set of data; *m* represents the sample size
*RMSE*	$RMSE=\sqrt{\frac{1}{m}{\sum_{i=1}^{m}\left(y_{i}-{\hat{y}}_{i}\right)}^{2}}$	
*MAPE*	$MAPE=\frac{100\%}{m}\sum_{i=1}^{m}\left|\frac{{\hat{y}}_{i}-y_{i}}{y_{i}}\right|$	

**Table 5:** Parameter optimization results.

Model Parameters	Optimal Parameter Value
max_depth	20
min_data_in_leaf	1
max_bin	256
num_leaves	170
bagging_fraction	1
bagging_freq	73
feature_fraction	1
lambda_l1	0.001
lambda_l2	0.001

**Table 6:** The comparison results of Bayesian-LightGBM on the training set and the test set.

Data Set	$R^{2}$	RMSE	MAPE
Training set	0.9800	0.0114	2.0325
Test set	0.9293	0.0356	5.5700

**Table 7:** The evaluation results of friction coefficient prediction model.

Models	$R^{2}$	RMSE	MAPE
RF	0.7464	0.0616	7.496
XGBoost	0.7921	0.0691	8.534
SVR	0.7885	0.0604	9.183
LightGBM	0.8981	0.0412	5.8120
Bayesian-LightGBM	0.9293	0.0356	5.5700

## Data Availability

Some or all of the data, models, and code generated or used during the study are available from the second author by request.
